# Uniparental Lineages from the Oldest Indigenous Population of Ecuador: The Tsachilas

**DOI:** 10.3390/genes12081273

**Published:** 2021-08-20

**Authors:** Tullia Di Corcia, Giuseppina Scano, Cristina Martínez-Labarga, Stefania Sarno, Sara De Fanti, Donata Luiselli, Olga Rickards

**Affiliations:** 1 Department of Biology, University of Rome “Tor Vergata”, Via della Ricerca Scientifica n. 1, 00173 Rome, Italy; martine@uniroma2.it (C.M.-L.); rickards@uniroma2.it (O.R.); 2 Department of Biological, Geological and Environmental Sciences, University of Bologna, 40126 Bologna, Italy; stefania.sarno@unibo.it (S.S.); sara.defanti@unibo.it (S.D.F.); 3 Interdepartmental Centre Alma Mater Research Institute on Global Challenges and Climate Change, University of Bologna, 40126 Bologna, Italy; 4 Department of Cultural Heritage (DBC), University of Bologna, Via degli Ariani, 1, 40121 Ravenna, Italy; donata.luiselli@unibo.it

**Keywords:** mtDNA, Y chromosome, Barbacoan, Native American, South America

## Abstract

Together with Cayapas, the Tsachilas constitute the oldest population in the country of Ecuador and, according to some historians, they are the last descendants of the ancient Yumbos. Several anthropological issues underlie the interest towards this peculiar population: the uncertainty of their origin, their belonging to the Barbacoan linguistic family, which is still at the center of an intense linguistic debate, and the relations of their Yumbo ancestors with the Inca invaders who occupied their ancient territory. Our contribution to the knowledge of their complex past was the reconstruction of their genetic maternal and paternal inheritance through the sequencing of 70 entire mitochondrial genomes and the characterization of the non-recombinant region of the Y chromosome in 26 males. For both markers, we built comprehensive datasets of various populations from the surrounding geographical area, northwestern South America, NW, with a known linguistic affiliation, and we could then compare our sample against the overall variability to infer relationships with other Barbacoan people and with other NW natives. We found contrasting patterns of genetic diversity for the two markers, but generally, our results indicated a possible common origin between the Tsachilas, the Chachi, and other Ecuadorian and Colombian Barbacoans and are suggestive of an interesting ancient linkage to the Inca invaders in Yumbo country.

## 1. Introduction

Our knowledge of the pre-colonial history of South America native peoples, which was formerly handed down by oral tradition, began to be recorded on written documents by the first chroniclers at the time of the conquest. Hence, it is characterized by several fragmentary and contradictory tales.

In this context, genetic investigation, which has become increasingly refined in recent years, have shown important insights not only on the first colonization and migrations events into the southern continent [1,2,3,4] but also on issues of historical and cultural interest for unraveling histories on a more local scale [5,6,7].

However, the origin of peoples who lived at the edge of the empires that dominated the Andes in the past centuries remained entrusted to the oral transmission for a longer time, hence the knowledge of their past is even more difficult to inquire [8,9]. The genetic studies centered on these regions, moreover, have to deal with further limitations due to small sample sizes or lack of uniformity in the resolution of genetic markers [6,7,8].

Our study focuses on the genetic history of a small Ecuadorian population, the Tsachilas, also known as the *Colorados*, that has several interesting reasons to be anthropologically investigated.

First, the Tsachìlas constitute, together with the Chachis (or Cayapa), the oldest population in the territory of Ecuador. They currently live in the Pichincha province in the Canton of Santo Domingo de los Colorados at the western foothills of the Andes. According to some historians, during the year 980 CE, the *Caras*, a population from the isthmus probably belonging to Chibcha linguistic family, conquered the Kingdom of Quito and over the next 200 years, occupied the entire region northwest of the current province of Pichincha, where the rivers Silanchi, Toachi, Blanco, and Caoní converge. During the Late Period, the *Caras* mingled with the natives of Quito and, over time, the Caras culture gave birth to the Yumbos, the ancestors of the current Tsachilas and Chachi people [10,11]. Tsachilas believe themselves to be descended from the people of the Andean highlands [12].

Second, the Tsachilas speak a Barbacoan language related to the languages of the Caranquis, the Pastos, and other groups in northern Ecuador and southern Colombia, which some linguists have included in a larger Chibcha-Paez family [13,14] although some others did not consider this affiliation plausible [15,16]. Louisa Stark [17] reports that Proto-Barbacoan languages split into the Chachi-Tsachilas branches in about 50 B.C.E. and that Chachi and Tsachilas remained a single language until they separated in about 1000 C.E. Before the arrival of the Incas in Ecuador, the Barbacoan language extended from the Guaytara River in Colombia to the Tungurahua province in Ecuador and spread down the central cordillera to near Quito. The linguists Curnow and Liddicoat [18,19] have suggested a family tree, subgrouping the Barbacoan languages from Colombia and Ecuador with Guambiano and Totoro, clearly forming a group, as do Cha’palaachi (or Chachi) and Tsafiqui, but that are not related to Paez languages although this hypothesis has been a matter of an intense debate between linguists.

Third, some historians have also found traits of cultural (but not linguistic) affinity between Yumbos and the Incas who occupied the area at the time of the northward expansion of *Tahuantinsuyo* [20] although much uncertainty remains about both the chronology and the nature of these relationships. During the period of the Spanish conquest, the Yumbos still resided in a territory closer to the slopes of the Andes Mountain range and to Quito, but they soon migrated lower, into the jungle area, due to violent eruptions of the Pichincha volcano in 1560, 1600, and 1690.

Based on the analysis of linguistic studies and the existing archaeological and ethnohistorical information, Lippi [21] proposed the hypothesis according to which the Yumbos separated from the Caranquis and the Panzaleos of the *sierra* and went to live in the tropical forest of Pichincha: the Tsáchilas (Colorados) of Sto. Domingo are, to some extent, the last descendants of the Yumbos and are probably of other ethnic groups from the western forests. Nowadays, their population comprises 2950 people, according to the last census [22], and they are organized into eight communities: Chiguilpe, El Poste, Peripa, Congoma, Otongo, Mapalì, Naranjos, Tawaza, and El Bùa. The Today, the Tsáchila economy is based on the staple crops of plantains, manioc, yams, cacao, peppers, maize, and rice. They also keep some domestic animals, but fishing is more frequently practiced, often with the use of poisons extracted from forest plants [23]. Similarly, we also know that the ancient Yumbo mostly cultivated plantains, manioc, corn, camote, and cotton plants, although there is no concrete documentation on the social organization or the economy in Yumbo country. They were skillful merchants that built interregional relations not only by trade, but also through the conquest of spaces and resources on a material and symbolic level [24].

At the time of the sampling mission, the Tsachilas were still quite isolated from the surrounding areas, and they have had only limited contact with the national culture and with non native people, probably retaining much of the original genetic variability.

In order to obtain a genetic perspective to integrate these archeological, ethnohistorical, and linguistic scenarios, this study focuses on uniparental markers: mitochondrial genomes (mtDNA) and the non-recombining portion of the Y chromosome.

The mitochondrial data for South American populations are mostly represented by control region sequences [25,26], which are useful for an evaluation of the distribution of frequency of the four major haplogroups, A, B, C, and D, across our area of geographic interest. We generated data of full mtDNA genomes to gain a better resolution of the haplotypes and to significantly improve insight into the population relationships that have been demonstrated by previous studies [27,28]. Relatively abundant data are available in the literature from the microsatellites and SNPs of the non-recombining portion of the Y chromosome [29,30,31] and indicate that the macro-haplogroup Q and its sub-lineages is by far the most represented across the native people from South America. We sequenced 23 Y-STR and 8 Y-SNPs to explore the Tsachilas variability and to evaluate it against other surrounding populations from the northwest part of the subcontinent.

We expect that these uniparental markers will help to clarify the relationships that the Tsachilas have with the Indigenous Chachi, who are currently living on the coast in the Esmeralda province [12]; whether their genetics match with their linguistic affiliation to the Barbacoan family; and finally, what the molecular analysis tells us about the findings of historians on the relationships between the Yumbo and the Inca. Our results indicate different genetic landscapes for the maternal and paternal data and provides a contribution from a molecular point of view on the theories of the origin of the Tsachilas people as well as on the linguistic debate and the knowledge of past events of peculiar anthropological interest.

## 2. Materials and Methods

### 2.1. Data Collection and DNA Extraction

**Figure 1:**
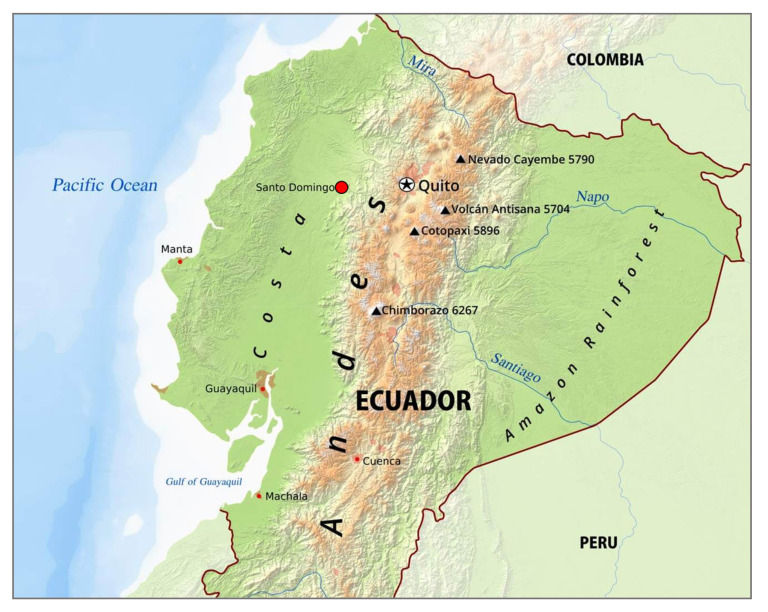
Map showing the reference location of samples investigated in the present study. The red dot indicates the city of Santo Domingo de los Tsachilas within the homonym canton.

Blood samples from 70 unrelated individuals were collected during the year 2000 during an anthropological mission conducted by Gianfranco De Stefano in the canton of Santo Domingo de los Tsachilas, Ecuador (Figure 1), from two Tsachilas communities named Chiguilpe and Congoma. Informed consent for the anonymous use of their data was obtained from all of the sampled subjects so that everyone consented to providing material for biological study. The study was approved by the Ethics Commission of the University of Rome “Tor Vergata”, and all procedures were undertaken in accordance with the Declaration of Helsinki on ethical principles. DNA was extracted using the “salting out” method [32].

### 2.2. mtDNA and Y Chromosome Genotyping

The full mtDNA genomes were amplified with 11 overlapping PCR fragments using a set of primers, and the Cycle Sequencing was performed using a set of 32 nested primers, as described in Torroni et al. [33]. The sequencing was performed by using an ABI 3100 sequencer with 4 capillaries. The DNA sequences were aligned using the software SeqScape 2.6 (Applied Biosystems, Waltham, MA, USA), according to the revised Cambridge Reference Sequence (rCRS) [34] and were manually revised for alignment errors. Subsequently, the major haplogroups were assigned using Haplogrep 2.2 [35], according to the nomenclature of the latest version of Phylotree Build 17 [36]. The hotspot sites at positions 16.182–16.193 and 16.519 and the indels 303–315 and 515–522 have been excluded from the statistical analyses. The 23 Y-STRs genotyping of all male individuals (29) was performed using the PowerPlex^®^ Y23 System (Promega, Madison, WI, USA). The amplified products were analyzed with an ABI PRISMR 3130 Genetic Analyzer (Applied Biosystems) following the recommended sequencing kit protocols and were then analysed with GeneMapperR ID Software v3.2 which, identifies an allele for each of the 23 loci. All the successfully genotyped individuals (26) were assigned to a haplogroup through a Bayesian inference on their Y-STR profiles [37], and only the haplotypes belonging to the native Q haplogroup (24) were used for the following analyses. We then tested eight selected Y-SNPs with a SNaPshot^TM^ Multiplex kit (Applied Biosystems) following the protocol described by Sevini et al. [38] to assign each haplotype to a Q sub-lineage, and we completed the haplogroup nomenclature assignment in accordance with Jota et al. [39].

### 2.3. Data Analysis

The calculation of the molecular diversity indices shown in Tables S1 and S2 [7,9,30,40,41], the estimation of the F_st_ distance matrices used for MDS analysis (φ_st_ for mtDNA haplotypes and R_st_ for Y-STRs), the shared haplotypes for both the mtDNA and Y chromosome data, and the AMOVA measures were performed with Arlequin v3.5 [42]. To visualize the distances between populations, a principal component analysis (PCA) was performed with mtDNA haplogroups frequency data by smart pcabelonging to the Eigensoft package [43] on a dataset of 53 populations from all over northern South America [6,7,9,25,40,44,45,46,47,48,49,50,51,52,53,54,55], shown in Table S3. The frequencies of the mitochondrial sub-lineages are shown in the table and pie chart in Table S4. For all of the haplotype-level analyses, two comparative datasets of both the mtDNA and the Y-STRs haplotypes have been used. The mtDNA dataset includes the 313 full mtDNA genomes listed in Table S5 from the northwestern area of South America (Peru, Ecuador, Colombia, and Bolivia), which were selected on the basis of the geographical proximity of these populations to our area of interest and on the availability of data regarding the linguistic affiliation [27,28,40,54,55,56,57,58]; moreover, the Y-STRs dataset includes 316 haplotypes for 14 loci listed in Table S6 over the same geographical area and belonging to the same language families where this kind of information was available [7,9,30,40,55]. We attempted to build two datasets that were as comparable as possible. Based on these datasets, a non-metric multidimensional scaling analysis (nMDS) was performed for both markers on the matrix of the pairwise distances values using Past software [59], and median joining (MJ) networks were built on the haplotypes aligned with the DNA alignment software v. 1.3.3.2 using Network v. 4.6.1.3 (Fluxus Technology Ltd., Clare, Suffolk, UK). In the MJ networks, we assigned different weights to the polymorphic sites according to the transition/transversion rates and to the recurrence of mutations to minimize homoplasy, as previously described [60]. The variance measures for AMOVA were calculated on the same dataset, assigning each population to a group first on the basis of language affiliation and then on geography (country). We considered the most represented and significant linguistic groups within the dataset (Barbacoan and Quechua), and we did the same with geography comparing Ecuadorian and Peruvian populations (Table S7). Moreover, for the Y-STRs data exclusively, we built a larger dataset that included 774 haplotypes from the same geographical areas described above to perform a cluster analysis centered on more frequent haplotypes in order to find some Y chromosome lineages that could identify episodes of male reproductive success over generations using the Star Cluster generation software as described in Balaresque et al. [61]. Finally, we estimated the time to the most recent common ancestor (TMRCA) of the unique relevant cluster using BATWING [62] with an exponential growth model, a generation time of 30 years [63], and a mutation rate of 0.022 per locus per generation for the microsatellites [64], and postprocessed it with R (http://cran.r-project.org (accessed on 28 January 2021)) to calculate the mean TMRCA values.

## 3. Results

### 3.1. mtDNA

We generated 70 novel sequences of complete mitochondrial genomes. All of the mtDNA profiles belonged to the main Native American haplogroups A2, B2, C1, and D1.

**Figure 2:**
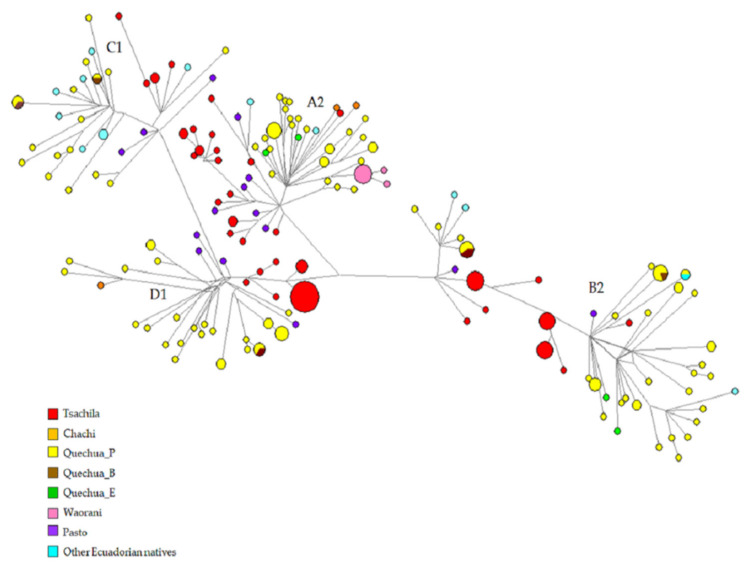
Median joining network for A2, B2, C1, and D1 haplotypes of mtDNA entire genomes (*n* = 313) among Peruvian, Bolivian, Colombian, and Ecuadorian populations color-coded by ethnicity/linguistic affiliation. The haplotypes are represented by circles with sizes proportional to the number of individuals and branch lengths proportional to the number of mutational steps. The Quechua populations are divided by country (Peruvian, Bolivian, and Ecuadorian Quechua are indicated by Quechua_P, Quechua_B, and Quechua_E, respectively).

Mitochondrial haplogroups B2 and D1 were the most frequent lineages in Tsachilas samples, as shown in Table S3, which also summarizes the haplogroup frequencies for 53 Native populations from all over northern South America. The entire mtDNA genomes also allows the identification of sub-haplogroup frequency in our Tsachilas sample, as shown in Table S4: it can be noticed that the most represented mitochondrial sublineage is A2ac, one of the ancient native lineages that is also detected in North and Central America and dated back to 15–12 Kya [28]. The PCA analysis based on the haplogroup frequencies shown in Figure S1 provides an overview of the distribution of these frequencies with the Tsachilas clustering with other Barbacoan populations, both Ecuadorian and Colombian (Chachi, Pasto and Cañar) and some Peruvian and Ecuadorian Quechua, while pointing away from the populations classified as Chibchan. We also tested the genetic variation at the level of complete mtDNA sequences by means of the molecular diversity indices (Table S1). The haplotype diversity (HD) of mitochondrial sequences is very high (0.99) and is comparable to some other agriculturalist northwestern populations whose entire mtDNA genomes are available [54]. Indeed, the Tsachilas are fishermen and slash-and-burn agriculturalists who settled in the forest in single-family houses [65]. To test the phylogenetic relationships at the individual level, we conducted a network analysis using the median joining network algorithm. The resulting MJ network is shown in Figure 2. No Tsachilas haplotype is shared with individuals from other ethnic groups (not even with Cayapa/Chachi), and this could be suggestive of an ancient separation between the two groups and long-lasting isolation from people of the surrounding area. We also consider a number of other interesting relatedness: in the A2 branches, they show a closer relationship with the Colombian Pasto, while the Chachi haplotypes cluster with Peruvian Quechua and one Tsachilas. The same occurs with B2, C1, and D1 lineages with most of the Tsachilas haplotypes being derived from Pasto nodes or being separated by only a few mutational steps. Among the B2 lineages, we also found a relationship with Ecuadorian natives from the Pichincha area (the original settlement area of Yumbo people), while the D1 Tsachilas lineages branch closer to the Peruvian and Ecuadorian Quechua.

**Figure 3:**
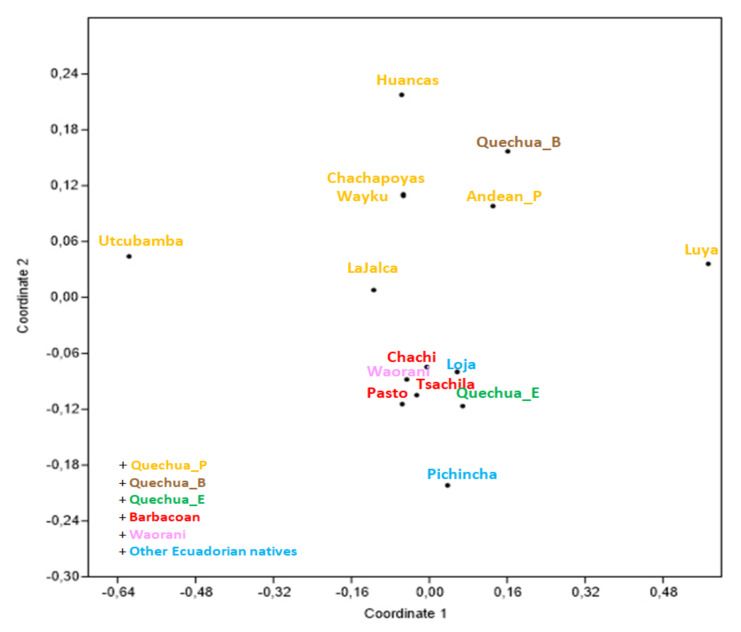
MDS plot based on φ_st_ genetic distances (stress value = 0.093) among 15 Ecuadorian, Peruvian, Bolivian, and Colombian populations.

Based on the same dataset that we used for the network (Table S5), we built a matrix of pairwise genetic distances (φ_st_), assigning each haplotype to one of the 15 Ecuadorian, Peruvian, Bolivian, or Colombian populations on the basis of ethnicity or language in order to elaborate a nMDS plot (Figure 3). It can be noticed that the Tsachila clearly point towards the other populations from Ecuador together with Pasto, a Barbacoan population from Colombia, while all of the other Peruvian and Bolivian Quechua occupy the upper part of the plot.

AMOVA analysis on the mtDNA data revealed a high degree of variance between the populations in the dataset (Table S7). We noticed that the Barbacoan populations are more homogeneous than the Quechua populations (7% vs. 10%) and that comparison between the two groups revealed a moderate proportion of variance between them. The analysis of variance based on geography grouping shows that the Ecuadorian populations are rather homogeneous between themselves.

### 3.2. Y Chromosome

The Y-STRs profiles revealed that 24 of the 26 male individuals belong to the native Q haplogroup, and the remaining two belong to Eurasian haplogroups (Table S8). We only considered the Q haplotypes for Y-SNPs characterization, and we found the native Q-M3 with one individual assigned to the Q-M19 sub-branchas unique lineage, which had previously only been described in Peruvian Amazon and Argentina [39,66,67]. The standard diversity indices calculated for the Y-STRs profiles indicates that the haplotype diversity of the Tsachilas (0.9) is similar to the average of the other Ecuadorian, Peruvian, and Colombian populations used as comparison (Table S2).

**Figure 4:**
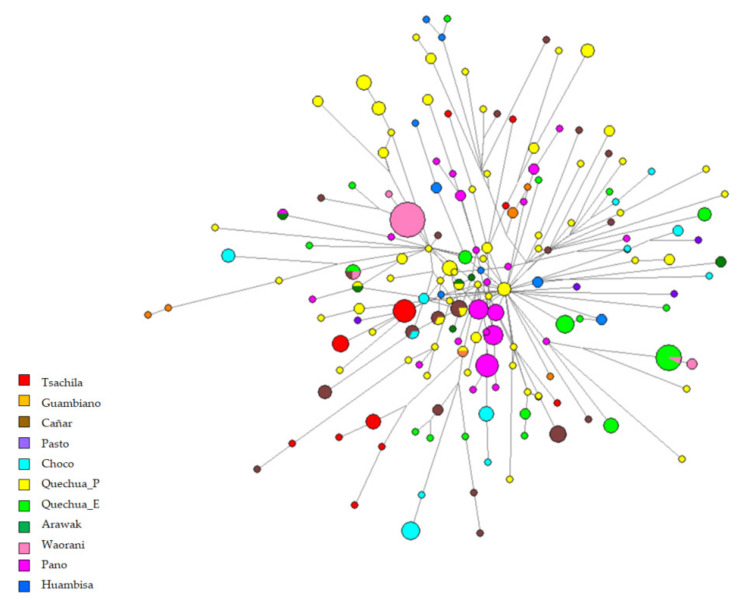
Median joining network for 14 Y-STRs haplotypes (*n* = 316) among Peruvian, Ecuadorian, and Colombian populations, color-coded by ethnicity/linguistic affiliation. The haplotypes are represented by circles with sizes proportional to the number of individuals and branch lengths proportional to the number of mutational steps. The Quechua populations are divided by country (Peruvian and Ecuadorian Quechua are indicated by Quechua_P and Quechua_E, respectively).

The network of Y-STRs haplotypes was only built with the Q-M3* haplotypes selected from the Y chromosome dataset since it was the unique haplogroup detected in our sample (Figure 4). The absence of shared nodes between Tsachilas individuals and other haplotypes is a common trait between the two uniparental markers. If compared tothe MJ network obtained from mitochondrial haplotypes, the phylogenetic relationships of male lineages highlighted a closer relationship between some Tsachilas and Peruvian Quechua nodes (including Peruvians of Inca kinship, published by Sandoval et al. [55]), while the other haplotypes show a relatedness with the Ecuadorian Cañar and Pasto and the Guambiano from Colombia (all affiliated with the Barbacoans).

**Figure 5:**
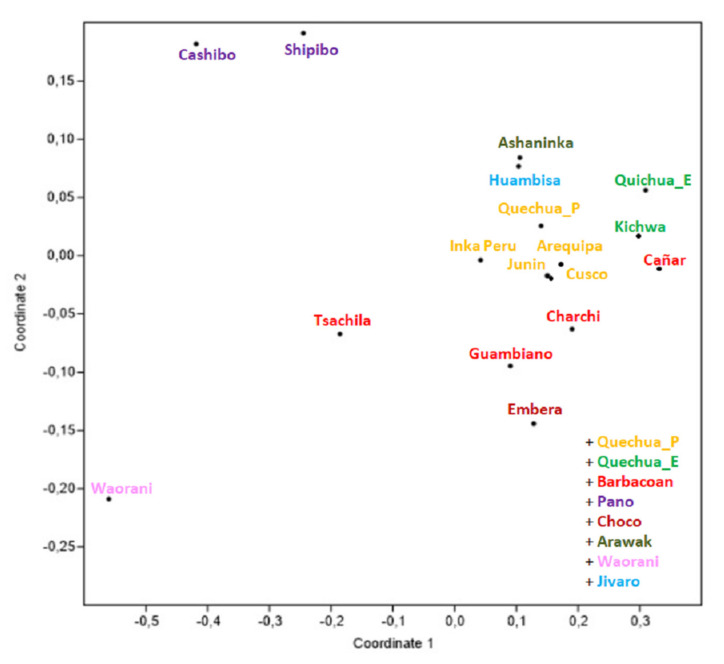
MDS plot based on F_st_ genetic distances (stress value = 0.079) among 17 Ecuadorian, Peruvian, and Colombian populations.

As was the case with the mitochondrial data, we used the same dataset for network and MDS analyses. In the MDS plot (Figure 5), it is noteworthy that along the first coordinate, the Tsachilas occupy an intermediate position between the Peruvian Pano and the Ecuadorian Waorani people as well as between all Quechua and Barbacoan populations, while along the second one, they point towards most of the Quechua populations and some of the Barbacoan populations. This is interestingly different from the maternal data, where the Tsachilas appeared to be more integrated into the Ecuadorian context.

If we look at the AMOVA conducted on the Y-chromosome data, we can notice that the variance between the Barbacoan populations is greater than in the mitochondrial data, and it decreases if we compare the Barbacoan and Quechua linguistic groups (Table S7). When considering geographic grouping, Ecuadorian populations are much more heterogeneous in Y chromosome data than in mitochondrial data, and in both cases (mitochondrial and Y-chromosome data), the variance between the Ecuadorian and Peruvian geographic groups decreases if we include the Tsachilas samples in the Ecuadorian populations.

Since the Y-STRs network suggested a hypothetical closer relationship between our Tsachilas sample and some Quechua, despite the absence of shared haplotypes, we employed a cluster analysis based on the microsatellite data of the Y chromosome following the method published in Balaresque et al. [61].

This method allows us to search for hypothetical recent episodes of male lineage expansion our area of interest (NW area of South America). In order to find such signals of lineage transmission (as descent clusters, DCs), our 24 novel Y-STRs haplotypes were entered into a database that included 775 Y-STRs profiles belonging to more than 20 populations from all over the northwestern area of the sub-continent, and we selected only 14 loci to include an adequate number of profiles. Following the same logical proceedings used in a previous study [9], we calculated the shared haplotypes and the frequency of each haplotype within the entire database, and we then ranked them by frequency. The purpose was to discard the unique haplotypes and to follow the most frequent profiles because these are the ideal candidates to be the potential “cores” for DCs. We only selected the frequent haplotypes that were found more than eight times in the dataset (1%). The resulting eight profiles were used as “core” haplotypes for the subsequent cluster analysis, and the correspondent DCs centered upon the cores were calculated, but only three of them included Ecuadorian samples. There were two of these three DCs (DC4 and DC5) that only comprised Waorani and some Kichwa, while only one, DC6, incorporated Tsachilas haplotypes (Table S9). The latter DC also caught our interest because it was the only Ecuadorian cluster including Peruvian profiles belonging to more than one population. In particular, DC6 comprised 70% of the Tsachilas haplotypes, 16% of the Peruvian haplotypes, and 5% of other Ecuadorian haplotypes. Despite the frequency values that indicate Ecuador as the area of major cluster expansion (particularly the Tsachilas population), the variance values instead suggest that the geographical origin of the cluster would be located in Peru (Table 1).

We also estimated the TMRCA using an exponential growth model in BATWING for the DC6 cluster to infer the possible timing for the origin of the cluster, and we obtained an interesting result dating the cluster back to about 1450 BCE, which was during the final period of the Inca empire.

## 4. Discussion

The Tsachilas seem to have preserved much of the ancient diversity that spread along the continent from north to south, which is mostly evident from the mitogenomes analysis. Indeed, they have retained some of the old mitochondrial sub-haplogroups, A2ac, A2k, B2b, D1f (Table S4), that likely arose in North or Central America prior to their early spread into the southern continent and probably arrived together with other founder haplogroups at the time of the first human entry into the subcontinent between 16.0 ka and 14.6 ka [28,68,69]. Interestingly, we also found one lineage restricted to Ecuador (B2b5a), which dates between 6.0 ka and 3.5 ka [28]. Unlike the mitochondrial data, the Y chromosome profiles do not allow sub-lineages with peculiar geographical distribution or age to be distinguished since all males belong to the same haplogroup Q-M3, unless the unique individual can be assigned to the Q-M19 sub-branch, which was previously only found in the Peruvian Amazon.

An old classification of South American languages [13,70] claims the Barbacoan languages as Tsafi’qui (distributed through southern Colombia and northern Ecuador) as a part of a bigger linguistic family called Chibcha-Paezan, which spread in Central American, Ecuador, and Colombia. However, later, a careful low-level linguistic reconstruction by other linguists did not find evidence of connections between these languages despite the popularity of this kind of link in classifying the South American languages [19]. If we look at the distribution of mtDNA haplogroup frequencies in a PCA plot (Figure S1), we should agree with this last reconstruction due to the marked distance between Tsachilas and other Colombian Chibchan, and, on the other hand, a clear proximity between the Tsachilas and Chachi seem to confirm the hypothesis of their common descent from ancient Yumbos.

We did not find any haplotype shared with other populations, indicating a high level of endogamy [71], even if the molecular diversity indices exhibit a good level of genetic diversity for both female and male lineages, and this could be explained by a fair level of gene flow between the different Tsachilas groups due to numerous inter-community marriages.

The haplotype-level analyses were also useful to clarify the relationship between the Tsachilas and the populations of the surrounding area, both Barbacoan and not, at a finer scale and we also useful to deepen the question concerning their hypothetical origin. The MDS analysis confirmed a genetic proximity with the Chachi (or Cayapa) on the maternal side (Figure 3). However, in the network, (Figure 2) we noticed that this relatedness was not as close as expected if we consider their supposed common ethnogenesis, as already shown in a previous study [65], which is probably due to the current existence of a linguistic barrier, which could have prevented bya long-time extensive intermixture between the two populations. Their languages are now mutually unintelligible although they are closely related, and their common past as ancient Yumbo could have been partially lost over generations through several decades of isolation.

The MJ network of the maternal lineages showed a closer relationship with the Pasto people (often two or three mutational steps), a population from Colombia whose language became extinct; they were probably linguistically affiliated to the Barbacoans together with Cara people, with both of them being of Andean origin, and this could support the oral tradition of Tsachilas regarding their own origin, although documentation is scarce [72]. However, the Y chromosome haplotypes seem to be more closely related to Andeans, as shown in MDS and network (Figure 4 and Figure 5), although a certain degree of genetic affinity with the Charchi, Cañare, and Guambiano is to be highlighted: they are all included in the South Barbacoan family tree created by Constenla [73] and Curnow and Liddicoat [18], together with Tsafi’qui and Cha’palaachi.

As other studies have previously showed [74,75], the genetic patterns of female and male lineages frequently diverge, as we can observe when comparing Tsachilas diversity against the genetic makeup of other Ecuadorian and non-Ecuadorian natives.

Generally, we found that the genetic structure was more influenced by the linguistic affiliation in mtDNA than in Y chromosome data, as it emerged from MDS (Figure 3, Figure 4 and Figure 5) and AMOVA (Table S7). The female lineages seem to have a higher potential to explain the original genetic structure of the first settlement of the area, and they generally show less genetic affinity with non-Ecuadorian populations if compared to male data, which is probably due to a lower degree of female mobility across the entire NW area of the subcontinent.

The increase of inter-ethnic marriages is documented in the recent history of Tsachilas, who were originally endogamic communities [24]. However, while we find traces of this phenomenon at a micro-geographic level on the maternal side of genetic inheritance, on the paternal side, we find more affinities between different populations if we look at our data in a broader linguistic or geographical context.

We wondered about the possible explanations for this difference. Assuming that the different resolution of the genetic markers analyzed (entire mitochondrial genomes and Y microsatellites) could have a role in the observed differences, we found that some authors reported that the basic Tsachilas social unit is the nuclear or extended patrilinear family and that between neighboring ethnic groups, the kidnapping or exchange of women was quite frequent [71], this could explain the higher homogeneity in mtDNA between neighboring populations.

The male data, on the other hand, indicate a greater similarity with non-Ecuadorian populations (mainly Peruvians), and we aimed to deepen the question with further analyses on the Y chromosome.

Since Y chromosome STRs seem to be informative for more recent contact effects between different populations, we used this marker to infer past Y chromosome lineage expansion events by patrilineal descendants. We found only one descent cluster thatincluded Tsachilas haplotypes among those selected according to the procedure described in the Data Analysis section. Interestingly, this spans from the western foothills of the Ecuadorian Andes to the Peruvian Andes and the highland regions of the Peruvian Amazon (Table S9), indicating that the primary haplotype (most likely Peruvian) may have had a considerable expansion and may have been inherited through numerous generations, transforming into a successful cluster dating back to about 1450 C.E.

These data are certainly intriguing since several archaeological studies indicate a possible relationship between the Inca and Yumbo during the northern expansion of the Tahuantinsuyo in the final stages of the Inca empire and a hypothetical certain degree of admixture between natives and invaders in Yumbo Country [20,76,77]. Although there is an uncertainty about the chronology and nature of these relationships, it is supposed that the Yumbos served as producers and traders of tropical goods that were highly valued by the Incas, and some authors have speculated that the Inca presence in Yumbo sites such as Palmitobamba could have started around 1534 after the fall of the Tahuantinsuyo, when Atahualpa was executed and the Quito nobles took refuge in Yumbo Country [77]. In this perspective, our results could support the hypothesis of the admixture mentioned above, although the Yumbo ethnicity is now extinct, and our investigations are necessarily indirect.

Summarizing, the molecular analysis leads us to confirm the Tsachilas as members of the large Barbacoan family, which extends from Colombia to Ecuador, according to linguistic family trees [18]. Particularly, we found signals of closer similarity with their ancient neighbors, the Cayapa (or Chachi), which formed part of the extinct ethnicity of the Yumbos, together with the Tsachilas. Moreover, we reported a possible interbreeding with Inca groups that once inhabited the territory occupied by the ancestors of the Tsachilas.

We believe that studies on full mitochondrial genomeconducted at higher molecular resolution s and with a higher number of Y-STRs and Y-SNPs may help in the future to disentangle the origins of the Barbacoan and Chibcha-Paez populations. In fact, high resolution molecular data integrated with linguistic and historical information will help to unravel questions regarding (i) the genetic affinities between the Barbacoan and the macro Chibcha-Paez language families; (ii) the timing of genetic diversification of the Proto-Barbacoan groups; and (iii) the degree of genetic affinity with Inca people and whether this could have eventually altered the genetic legacy of present-day Ecuadorian natives.

Nowadays, Tsachilas communities are little larger than at the time of the sampling mission, but they are also more integrated into the urban population and admixed with people of non-native ancestry, so the present study represents one of the last pictures of the native genetic landscape of pre-Columbian Ecuador.

## Figures and Tables

**Table 1:** Principal features of Descent Cluster 6 (DC6), including the number of microsatellite profiles, the countries, and the populations where the cluster expanded, the maximum values of frequency and variance, the TMRCA estimates, the growth generational growth rate (α), and the period that the cluster dates back to.

N	18
Countries	Ecuador, Peru
Populations	Tsachila, Charchi, Huambisa, Chachapoya, InkaD
Max frequency	83% (Ecuador)
Max variance	0.095 (Peru)
TMRCA (95% CI)	571 (90–3100)
Alpha	0.0217
Period	1450 CE

## Data Availability

mtDNA genome sequences in FASTA format of the 70 individuals have been deposited in the NCBI GenBank under accession numbers MZ397809-MZ397878.

## Supplementary Materials

The following are available online at https://www.mdpi.com/article/10.3390/genes12081273/s1, Figure S1: PCA plot, Table S1: General information and diversity data for Tsachila sample, Table S2: Diversity indices values for populations included in Y-chromosome dataset, Table S3: Frequency of mtDNA haplogroups in 53 populations from northern South America, Table S4: Frequency of mtDNA sub-haplogroups, Table S5: mtDNA dataset, Table S6: Y-STRs dataset, Table S7: AMOVA results; Table S8: Y-STRs and Y-SNPs profiles of the 26 male individuals, Table S9: Y chromosome haplotypes included in Descent Cluster 6 (DC6).
