# Supplementary figures and images for: Uniparental Lineages from the Oldest Indigenous Population of Ecuador: The Tsachilas
Source: Genes (Basel). 2021 Aug 20;12(8):1273. doi: 10.3390/genes12081273 (PMC8391833; doi:10.3390/genes12081273)

PC2: 26,8 %

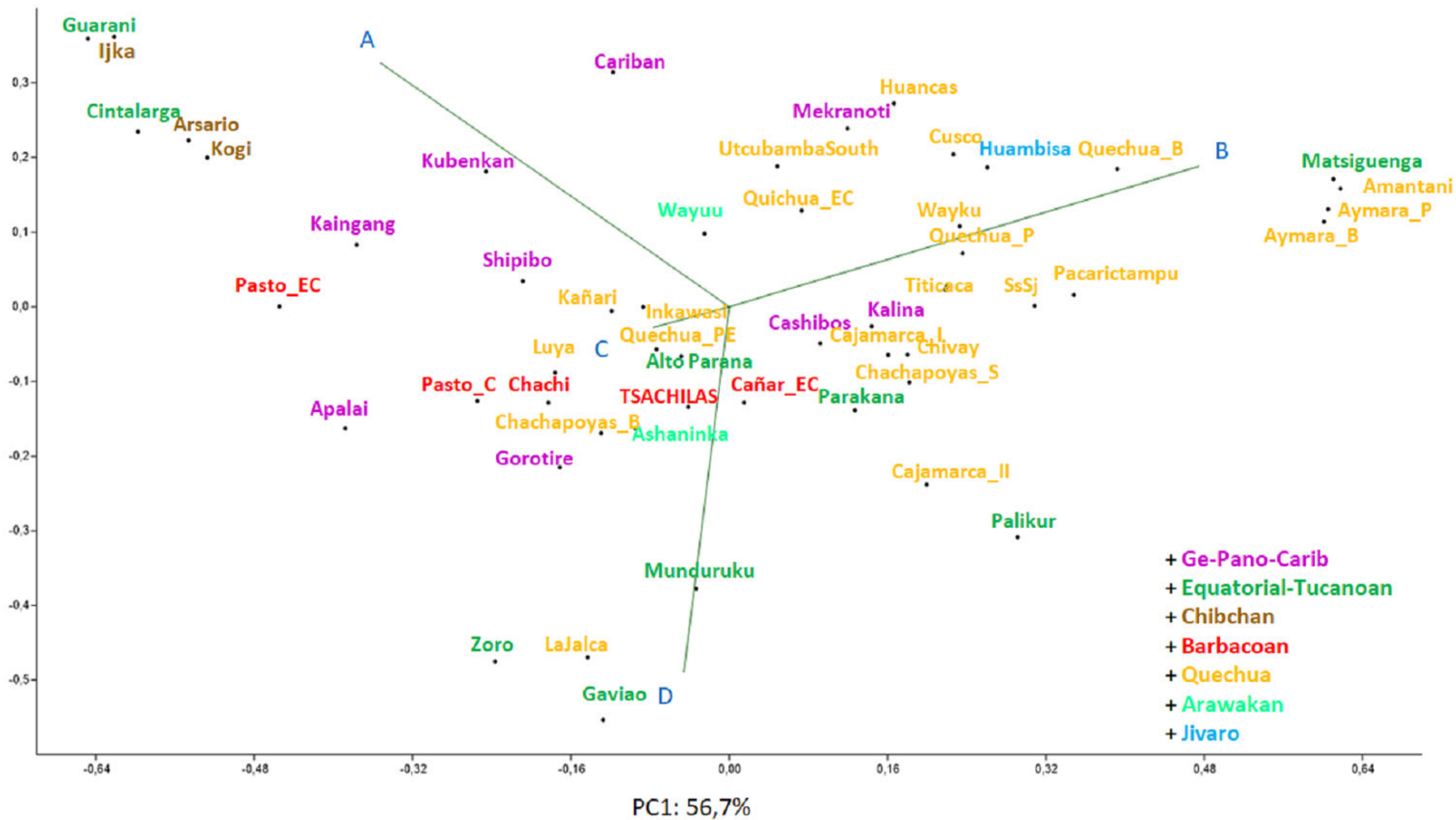

Supplement: Supplementary file 1 [file genes-12-01273-s001.zip › SUPPLEMENTARY/FIGURES/Figure S1.pdf]
